# Using EMS Dispatch to Trigger STEMI Alerts Decreases Door-to-Balloon Times

**DOI:** 10.5811/westjem.2015.4.24248

**Published:** 2015-04-21

**Authors:** Justin C. Stowens, Seema S. Sonnad, Robert A. Rosenbaum

**Affiliations:** * Christiana Care Health System, Department of Emergency Medicine, Newark, Delaware; † Christiana Care Health System, Value Institute, Newark, Delaware; ‡ State of Delaware Office of Emergency Medical Services and Health Preparedness, Christiana Care Health System, Department of Emergency Medicine, Newark, Delaware

## Abstract

**Introduction:**

We sought to determine the potential reduction in door-to-balloon time (DTB) by allowing paramedics to perform prehospital ST-Elevation Myocardial Infarction (STEMI) notification using brief communications via emergency medical services (EMS) 9-1-1 dispatchers as soon as they saw a STEMI on 12-lead electrocardiogram (EKG). Our hypothesis was that earlier cardiac catheterization lab (CCL) activation would improve overall DTB and avoid delays arising from on-scene issues or the time required to deliver a full report.

**Methods:**

The study setting was a single suburban community teaching hospital, which is a regional percutaneous coronary intervention (PCI) center with more than 120,000 Emergency Department (ED) visits/year and is serviced by a single tiered-response, advanced life support (ALS) paramedic-level agency. STEMI notifications from July 2009 to July 2012 occurred by either standard direct EMS-to-physician notification or by immediate 9-1-1 dispatch notification. In the 9-1-1 dispatcher-aided notification method, paramedics were asked to provide a brief one-sentence report using their lapel microphones upon immediate realization of a diagnostic EKG (usually within 1–2 minutes of patient contact). This report to the 9-1-1 dispatcher included the patient’s sex, age, and cardiologist (if known). The dispatcher then called the emergency department attending and informed them that a STEMI was being transported and that CCL activation was needed. We used retrospective chart review of a consecutive sample of patients from an existing STEMI registry to determine whether there was a statistically significant difference in DTB between the groups.

**Results:**

Eight hundred fifty-six total STEMI alert patients arrived by EMS during the study. We excluded 730 notifications due to events such as cardiac arrest, arrhythmia, death, resolution of EKG changes and/or symptoms, cardiologist decision not to perform PCI, arrival as a transfer after prior stabilization at a referring facility or arriving by an EMS agency other than New Castle County EMS (NCC*EMS). Sixty-four (64) sequential patients from each group comprised the study sample. The average DTB (SD) for the standard communication method was 57.6 minutes (17.9), while that for dispatcher-aided communication was 46.1 minutes (12.8), (mean difference 57.6-46.1 minutes=11.5 minutes with a 95% CI [6.06,16.94]) p=0.0001. In the dispatcher-aided group, 92% of patients (59/64) met standards of ≤60 minute DTB time. Only 64% (41/64) met this goal in the standard communication group (p=0.0001).

**Conclusion:**

Brief, early notification of STEMI by paramedics through 9-1-1 dispatchers achieves earlier CCL activation in a hospital system already using EMS-directed CCL activation. This practice significantly decreased DTB and yielded a higher percentage of patients meeting the DTB≤60 minutes quality metric.

## INTRODUCTION

Cardiac catheterization is the preferred treatment for patients suspected of having an ST-segment elevation myocardial infarction (STEMI). The epidemiology of acute coronary syndrome (ACS) and STEMI is staggering. With 1,680,000 estimated ACS discharges yearly and >500,000 estimated STEMI events in the United States yearly the impact of improving treatment of this disease cannot be overstated.^1^ At the time of data collection for this publication, the Ameruican College of Cardiology (ACC) guideline considered a “door to balloon” (DTB) of less than 90 minutes the treatment goal.^1^ Although the guideline is nearly a decade old, compliance remains difficult for many hospital systems.^2^ Our hospital system strives for a DTB metric of less than or equal to 60 minutes. A recent review by Camp-Rogers concluded that of eight approaches associated with reduced DTB, only two, including emergency medical services (EMS) activation of the cardiac catheterization lab (CCL), had sufficient evidence to support causality.^3^ This review found 18 studies examining EMS CCL activation, all associated with decreased DTB. The different methods of EMS CCL activation including wireless transmission from the paramedic monitor, cell phone transmission, and activation of the CCL with complete bypass of the emergency department (ED). They were all associated with decreased DTB. Bradley et al. reports a 15.8 minute decrease on average from their 2005 review for all types of prehospital activation.^4^ Concern about improper activation of the CCL is the major reason for incomplete adoption of EMS activation protocols, despite reported false positive rates below 10%.^3^,^5^–^8^ This paper describes a cost-free method for further streamlining and implementing EMS activation of the CCL for STEMI patients in a system already using EMS activation.

### Importance

The U.S. healthcare system currently struggles with providing efficacious care cost-effectively. The ACC/American Hearth Association guidelines at the time of data collection, now backed by Medicare and Medicaid performance standards, evaluate hospital systems’ abilities to meet a 90-minute DTB standard with the risk of decreased reimbursement facing systems that consistently exceed this target. For hospital systems already using prehospital CCL activation, any cost-free modification that could decrease DTB times without sacrificing the quality of patient care should be evaluated and adopted whenever possible.

### Goals of this Investigation

**Figure 1:**
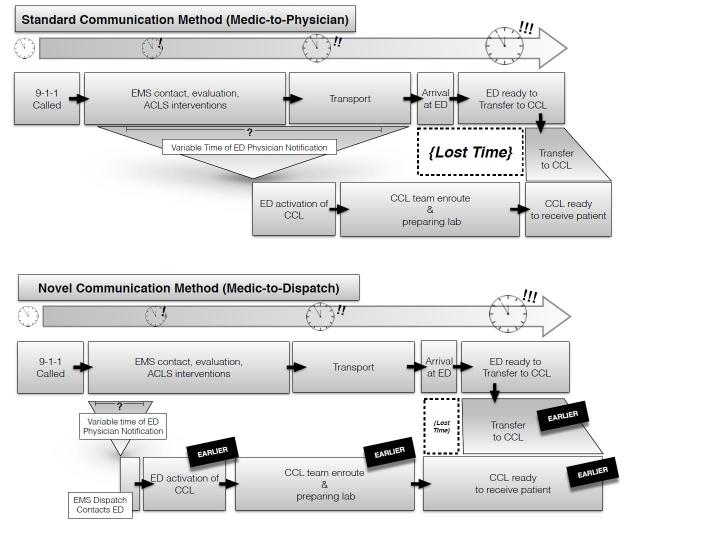
Comparison of standard and novel ST-elevation myocardial infarction notification methods. *EMS,* emergency medical services; *ACLS,* advanced cardiac life support; *ED,* emergency department; *CCL,* cardiac catheterization lab

We recognized that our prehospital system has relatively short patient contact times (20–30 minutes) and transport times typically are less than 10 minutes. Notification during patient interventions or transport may not provide sufficient time to fully prepare and staff the CCL with an interventional cardiologist. We sought to maximize the notification time window. This would permit more of the process of CCL team arrival and preparation to occur parallel to EMS evaluation, treatment and transport, allowing seamless transfer from the ED to the CCL and a greater proportion of DTB ≤60 minutes (Figure 1).

We hypothesized that under the prehospital STEMI alert model, bypassing discussion and lengthy notification with hospital personnel, DTB would be decreased significantly by earlier CCL activation. This advance notice would allow for more of the CCL preparation including arrival of personnel and an interventional cardiologist to occur parallel to EMS packaging and transport of their patient. Our goal was to demonstrate that this single intervention could decrease DTB by an average of 10 minutes.

## METHODS

### Study Design

This was a retrospective chart review of an existing STEMI registry comprising a consecutive sample of patients presenting to the ED between July 2009 and December 2012. The existing STEMI registry was compiled by trained research nurses in an ongoing fashion. They were blinded to the hypothesis of any ongoing studies, and data collection was begun prior to the generation of the research hypothesis. The same research nurses also applied the predefined inclusion and exclusion criteria to cases as they occurred to create a separate databank of consecutive cases for inclusion. This study relied on only one additional data abstractor (primary author) and they were trained in data abstraction prior to abstraction. This additional data abstractor, while not blinded to the study hypothesis, further excluded patients whose data collected by the initial abstractors were incomplete. They did not have the ability to add any additional cases. We defined all variables prior to abstraction, and the research nurses used abstraction forms. There was no inter-observer reliability testing performed. However, ongoing review of research nursing performance monitoring occurs in an ongoing fashion within the institution.

The majority of patients in our catchment area are served by a single, tiered-response, advanced life support (ALS) agency, New Castle County Paramedics (NCC*EMS). This agency transports over 95% of the ALS patients arriving at our facility, while almost all basic life support (BLS) calls are transported by the regional fire department-based responders. NCC*EMS employed approximately 100 paramedics at the time of this study. All paramedics are nationally registered (NR-EMT-P) and undergo field training certification that lasts 8–24 weeks (depending on prior experience) during which time they manage patients under the supervision of a field training officer (FTO). Beyond the NR-EMT-P level of electrocardiogram (EKG) training, 20% of the yearly in-service education is focused on STEMI recognition and mimics. Monthly quality assurance newsletters are distributed highlighting difficult EKGs. Individual cases of failed STEMI recognition are reviewed both formally with the medics involved and the medical director, as well as via distribution of the EKG with salient teaching points to the entire service. On average, the system has 1–3 occurrences per year where medics fail to recognize a STEMI.

In our system, prior to June 2009 NCC*EMS contacted the ED and gave a full report to an ED attending. This included the patient’s EKG findings, vital signs, and overall clinical picture as part of the standard pre-hospital radio notification (medic-to-physician approach). The notification proceeded from the ED attending to the clerical staff who would activate the CCL team. The paramedic’s work environment often is chaotic and filled with barriers to completing even basic assessments. It was not uncommon for notification between medic and emergency physician to be delayed until the patient was moved to the controlled environment of the ambulance. Paramedic decision to implement intravenous (IV) access and treatment on scene may further delay a request for CCL activation. At the receiving facility, it is not uncommon for a busy emergency physician also to experience delays getting to the radio. Other reasons for delay varied but included the CCL team had not yet arrived, delay of cardiologist, or no available operating table in the CCL due to cases in progress.

In July 2009 our system worked cooperatively with NCC*EMS and began allowing notification of a STEMI to reach the emergency physician via NCC*EMS Dispatch. Instead of spending the time to give the entire report along with STEMI notification, NCC paramedics were instructed to give one sentence to their EMS dispatchers that consisted of the words “STEMI notification” or “Heart alert” with the patient’s age and gender along with the name of the patient’s cardiologist, if known. The paramedics communicated STEMI status using a lapel microphone at the patient’s side immediately after obtaining and interpreting a diagnostic EKG and prior to any further intervention. The NCC dispatchers then made a direct call on an existing, dedicated landline phone connection. This connection from paramedic through dispatch to the receiving facility took 60–90 seconds from start to finish allowing activation of the CCL in 1–2 minutes from the acquisition of the EKG. Emergency physicians were instructed that the dispatcher would have no other information, and that they should act on these requests for activation of the CCL (medic-to-dispatch approach).

NCC*EMS is a high performance ALS service accredited by the Commission on Accreditation of Ambulance Services (CAAS). NCC*EMS was an early adopter of prehospital 12-lead EKG. NCC paramedics are trained to perform 12-lead EKG early in the assessment of any patient who they suspect could be having acute coronary syndrome (ACS), including complaints of chest pain and other angina equivalents. NCC paramedics perform their own interpretation of the 12-lead EKG and request activation of the CCL on patients with appropriate presentation and EKG changes meeting STEMI criteria delineated by the ACC. The LifePak^®^ monitors used by NCC*EMS also provide a computer interpretation that can help alert the paramedic to an abnormal EKG. The study intervention sought to capitalize on this early diagnosis by having paramedics immediately notify dispatch as soon as they obtained a diagnostic EKG.

Patients were assigned to a group for study analysis based on whether the CCL was activated via the new “medic-to-dispatch” approach or by a traditional medic-to-physician conversation. Our observational study capitalized on the ongoing use of the original medic-to-physician procedure parallel with use of the new medic-to-dispatch relay notification route to CCL activation beginning July 2009.

As the new procedure was made available and medics were trained to activate the CCL via EMS dispatch, some medics mistakenly continued to activate the CCL via the antiquated method. The original route of speaking to an emergency physician directly was left in place intentionally as the lines of communication between paramedic and physician needed to remain open for all other care direction. As paramedics continued to use the old notification pattern after July 1, these patients were captured as a control group for the group of patients for whom the medic-to-dispatch route of CCL notification occurred. All other aspects of patient care and progression through the hospital system remained the same between the two groups. Over the subsequent three years the number of patients for whom the medic-to-physician communication route was implemented decreased and the number of medic-to-dispatch patients increased. Per EMS administration there was no identifiable group of paramedics that routinely chose one route of communication over the other.

We selected the patients tagged for the chart review sequentially from this time period to overcome any bias that may have otherwise occurred from sampling at any particular time period during a period of nationally decreasing DTB. We also compared the two groups for demographic and pathologic co-morbid conditions (Table 1). There were no significant differences between groups.

The study protocol was reviewed and approved by the institutional review board. As this was a retrospective chart review, the institutional review board waived informed consent.

### Setting

Christiana Hospital is a 913-bed, Level 1 trauma center, located in Newark, DE. It is the only hospital of its size with Level 1 designation and CCL capabilities between Baltimore and Philadelphia. The ED sees >120,000 patients per year. The prehospital care system consists of county-sponsored ALS transport, BLS- trained fire department response, and hospital-based critical care transport. Paramedics transport between 400 and 500 non-transfer STEMI/NSTEMI patients to Christiana on average per year.

### Selection of Participants

We selected participant’s charts for review for inclusion in the study if they presented to the ED at Christiana Hospital between July 2009 and December 2012 and were subsequently taken to percutaneous coronary intervention (PCI).

### Inclusion Criteria

All included patients were prehospital STEMI alerted patients transported by EMS from the field and taken to CCL for PCI at Christiana Hospital between July 2009 and December 2012 directly from the ED. We selected the study group from a consecutive series of patients who arrived at our facility and were diagnosed with STEMI. Patients had to have arrived by NCC*EMS to be eligible for consideration. The patient had to have a STEMI on prehospital 12-lead EKG and CCL activation must be initiated by EMS report whether by standard radio contact or the medic-dispatch approach. STEMI diagnosis was not disputed and the patient moved to the CCL without delay for further diagnostic testing. All patients in the study group had angioplasty performed to allow evaluation of DTB time.

### Exclusion Criteria

We excluded patients if they arrived by any EMS agency other than NCC*EMS. We did not enroll patients under age 18, patients who arrived as transfers from other facilities, patents whose STEMI occurred after ED arrival or who were already inpatients when their STEMI occurred. Other exclusion criteria included receiving thrombolytics prior to PCI or any documented clinical reason for delay in the decision to proceed with PC, I including possible confounding diagnoses requiring testing, cardiac arrest or arrhythmia requiring intervention, respiratory failure requiring intubation, balloon pump insertion, or delays in patient consent for religious, social, or other personal reasons. We also excluded patients enrolled in other clinical trials.

### Data Collection and Measurements

For the study time period we retrieved demographic information, including age, sex, ethnicity, presence of selected cardiac comorbidities such as hypertension, diabetes mellitus, hyperlipidemia, prior PCI, atrial fibrillation, congestive heart failure, or known coronary artery disease, on all patients included in the chart review (Table 1). The DTB time, the arrival-to-PCI time, the diagnostic EKG-to-dispatch notification time, and the time-to-ambulance-ready time all were recorded for both groups and the averages calculated for comparison with determination of confidence intervals and p-values. Initial power analysis suggested that a minimum of 64 charts from each group would be needed obtain statistical significance for an effect size of a 10-minute difference in DTB time between groups (80% power for a significance of 0.05).

### Analytical Methods

We conducted hypothesis tests for differences between groups using Mann-Whitney tests for the differences in DTB time and using chi-square tests for the proportion of patients achieving DTB time of less than or equal to 60 minutes. Sub-analysis was also performed to control for a possible temporal bias, as DTBs generally decreased over the study period. We also performed linear regression on several covariates and created a multivariate model. False positive rates were not analyzed in this study as there is concurrent research in progress at our institution including the same time period; therefore, they were not included in our data set.

## RESULTS

**Figure 2:**
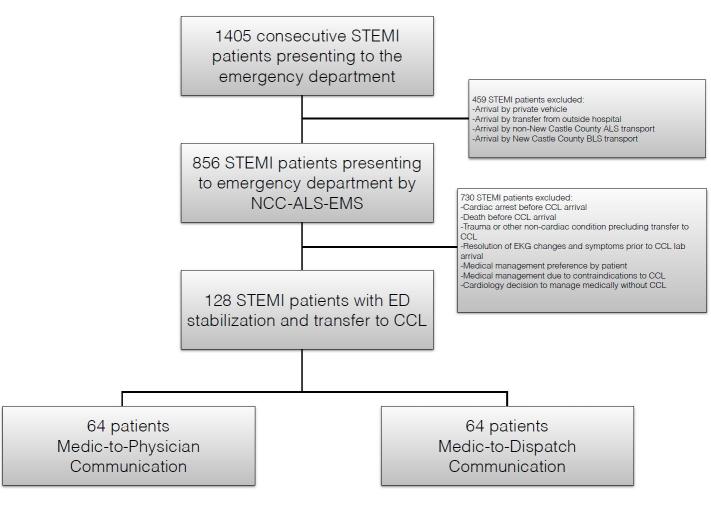
Enrollment flow chart with exclusions. *STEMI,* ST-elevation myocardial infarction, *NCC-ALS-EMS,* New Castle County-advanced life support-emergency medical services, *ED,* emergency department; *CCL,* cardiac catheterization lab; *BLS*, basic life support; *EKG,* electrocardiogram

A total of 1,405 STEMI notifications occurred during the study period; 856 notifications arrived by EMS. We excluded 730 notifications due to confounding events such as cardiac arrest, arrhythmia, death prior to PCI, resolution of EKG changes and/or symptoms, cardiologist decision not to perform PCI, prior stabilization at a referring facility, or because the patient was not transported by the EMS agency involved in the study, NCC*EMS. Of the remaining patients, we performed analysis of 64 sequential patients in each group (Figure 2). The average DTB for the standard communication method was 57.6 minutes (SD 17.9). The 9-1-1 dispatcher-aided communication average DTB was 46.1 minutes (SD 12.8). The difference between the two groups was an average of 11.5 minutes (95% CI [6.06,16.94], p=0.0001). In the 9-1-1 dispatcher-aided group 92% (59/64) met the metric of ≤60 minute DTB. Only 64% (41/64) met this goal in the standard communication group (p=0.0001). This decrease in DTB was consistent with prior reports of decreased DTB through various methods of EMS CCL activation.^4^

**Figure 3:**
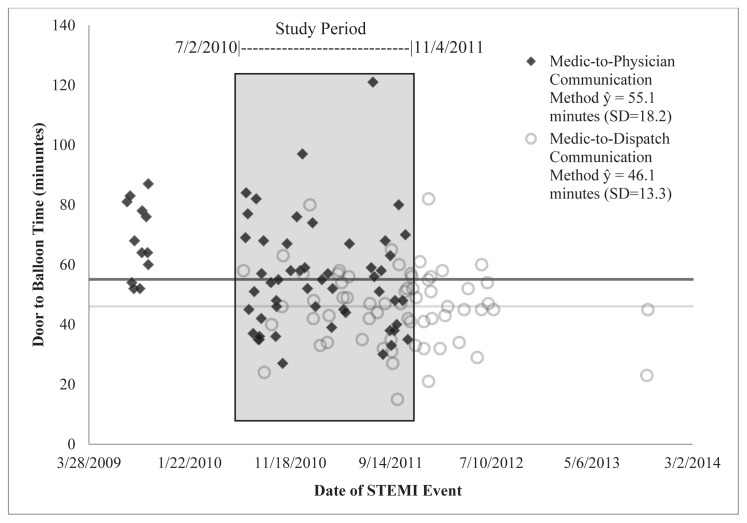
Door-to-balloon time over the length of the study. The shaded area reflects observations included in the analysis. *STEMI,* ST-elevation myocardial infarction

To determine if the treatment analysis was instead capturing a temporal effect of an ever-improving system, we conducted a sub-analysis on the observations between July 2, 2010 and November 4, 2011. These dates represent the first new method observation (7/2/2010) and the last standard method observation (11/4/2011). Figure 3 shows a scatterplot of DTB times over the length of the study. The shaded area shows the observations included in the analysis. The average DTB (and standard deviation) time for the standard communication method was 55.1(18.2) minutes and the average time for the dispatcher-aided method was 46.1(13.3) minutes, with an average difference of 9.0 minutes. Mann-Whitney testing showed that this difference was significant (p=0.0159). When comparing the percent of patients with a DTB of less than or equal to 60 minutes, the standard method had 71% (37/52) while the new method had 91% (30/33). This was a significant difference (p=0.0298). This group consisted of 85 patients. This sub-analysis demonstrates that when a possible temporal effect is controlled for, the difference of the effectiveness of the two methods is maintained and is still significantly present.

**Figure 4:**
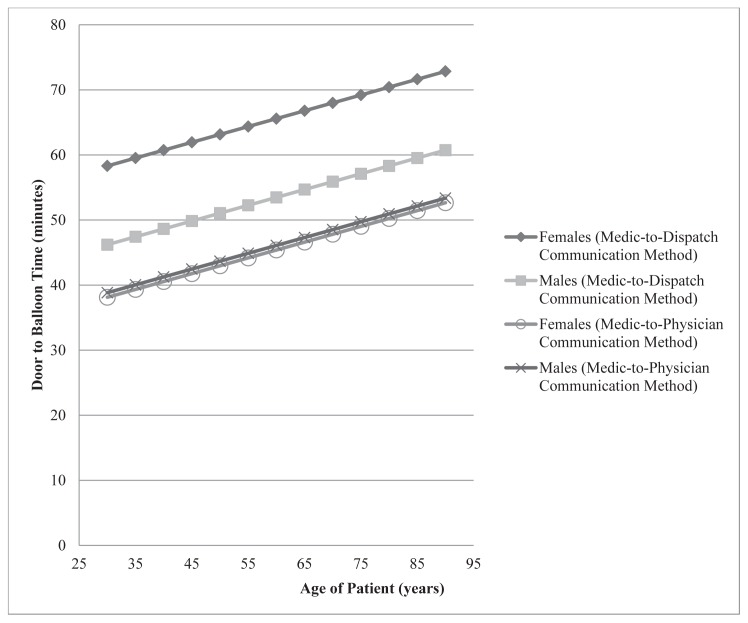
Logistic regression to predict door-to balloon time vs. age by sex and notification method.

The only significant predictors of DTB time, when looked at separately, were age, sex and congestive heart failure (CHF). We included these covariates in a linear regression model along with an interaction effect for treatment and sex. CHF was not significant in any multivariate models and was removed. The final multivariate model predicting DTB time from treatment, age, sex and a treatment* sex interaction showed that all predictors were significant and including these variables significantly improve the reduced models. In this model, the DTB difference (SE) is greater for females, 20.2 (4.8) minutes, than males, 7.4 (3.2) minutes. Figure 4 illustrates this difference and notably shows that age, regardless of sex, increased DTB time. Multivariate logistical modeling predicting patients with more than 60 minutes DTB time with controlling for covariates showed that there were no other significant predictors of the binary outcome other than the method of activation (p=0.0004) and patients with prior catheter without stents (p=0.0478). The dispatch-aided method reduced the relative likelihood of DTB being greater than 60 minutes by 86% (95% CI [59%–95%]). Prior catheterization, while significant, may not be a reliable indicator of longer DTB time with a low count of observations (Table 1), which is reflected in its wide confidence interval (1% to 2,800% increase). In all these models the treatment effect remains significant (p<0.001), so even when we controlled for variables that correlated with DTB independently, there is still a prominent decrease in DTB associated with the dispatcher-aided notification method.

The new procedure used only existing equipment so no equipment training was needed or costs incurred. To change paramedic practice pattern, a memo from EMS leadership was distributed to all paramedics describing the reasoning and details of the new protocol. Following the initiation, frequent communications from the medical direction or command staff were circulated through EMS platoons as a reminder of the process. Reports had no reliability issues that are sometimes associated with new technology such as WiFi or cellular transmission of EKG. This notification was rapid enough that it avoided patient care interruption, allowing acceptance by paramedics. The communication was brief, simple and consistent, allowing it to be easily relayed and not being overly burdensome to dispatch staff. ED staff members were well informed of the limit of information that would come to them from the dispatcher and that as a third party, the dispatcher would not have anything other than the brief communication to report. Because our facility had an existing acceptance to activate the CCL based on paramedic report/request, there was a willingness to accept this earlier request and activate based on this brief communication.

## DISCUSSION

DTB time reduction strategies are an important metric for STEMI-receiving facilities, and results presented here are consistent with the prior research included in the review by Camp-Rogers showing EMS to be a valuable tool to decrease DTB.^3^ The nature of the benefit of early notification may be multi-factorial. Earlier notice initiates the process and seems to be the most important factor. The brief report prevents any risk of important information delivery being delayed or misinterpreted if included in the full body of the paramedic report. The brevity of the report also insures rapid initiation of the request, which could be delayed if the physician listened to the entire report before initiation of the STEMI alert request. It should not be overlooked that the EMS dispatcher is unable to provide any “discussion” with the physician as to the details of how the patient met criteria for CCL activation. This removed any time used in discussion between the receiving doctor and the paramedic.

Our hospital and the paramedic agency involved have worked cooperatively for many decades. Our ED physicians and cardiologists have a high level of confidence in NCC*EMS with a willingness to accept some false activations. False activations by EMS have been examined in the literature, although few definitive conclusions or recommendations have been drawn. Some of the frequently cited studies by Camp Rogers found a false activation rate by EMS of 8%, while Garvey reports a 6% false activation rate and Lee reports 8.3%.^3^,^5^,^6^ There are similar false activation rates by emergency physicians reported in the literature. Youngquist reports 8.0%, Feldman reports a 93–95% accuracy for both emergency physicians and paramedics.^7^,^8^ With similar numbers reported throughout the literature it will still remain up the collaborative efforts between cardiology and emergency medicine at each individual institution to agree on an acceptable false positive rate.^9^,^10^ It is the belief of the authors that this tolerance of false positive activation is critical to allow an EMS-directed intervention, such as this one, to work to maximum benefit and achieve individual patient and system-wide benefits. In our system, feedback from the ED and cardiology on both false activations as well as missed activation opportunities are brought to the EMS medical director at monthly meetings. The EMS medical director then meets with the paramedics individually to review the case. The system has also employed a “STEMI coordinator” who provides feedback to the paramedics, usually before the end of their shift. Paramedics are also refreshed on EKG interpretation as part of their yearly educational curriculum. These benefits of rapid EMS recognition, notification, and transport to appropriate destinations will become even more important as new metrics such as first medical contact to balloon time becomes the standard.

Based on education provided to NCC*EMS regarding the time benefits of using the dispatch notification process, the use of the new process is now a widely accepted clinical practice in our system. Future questions to be followed include monitoring for an increase in the false activation rate of our CCL and if these shorter DTB times continue with regular use of the new activation pattern. A potential positive with this approach is the possibility that our system will be able to more frequently use direct transport of the patient from EMS to the CCL with the ability to bypass the ED. Recent research has suggested this may be a viable approach to further decrease DTB by greater than 30 minutes.^10^,^11^ A barrier to that implementation has been variability in the time for the CCL team to arrive and have the CCL ready for the patient. We hope that with regular advanced notification we will achieve consistent availability of the CCL team prior to patient arrival and that the use of direct EMS-to-CCL transportation may add to the improvements already seen with this intervention.

Our analysis also shows a direct correlation between DTB and both age and sex, with longer DTB being associated with both the elderly and women (Figure 4). The reasons for this were not obvious from the data but it might be hypothesized that the simple act of moving a more elderly patient to the hospital stretcher, recording medications, consenting for the procedure, assuring no contraindications to performing the procedure and gaining IV access may have increased their DTB. It may be that the elderly are a sicker population requiring more time for stabilization of STEMI-associated morbidity (intubation for respiratory failure, for example). Or it may simply be harder to pass the catheter wire though more atherosclerotic vessels.

The difference between genders is also unclear. It is known that women generally present for STEMI at an older age and therefore the sex may be an association and not a true causality of longer DTB.^12^ However it is also known that women present later to the hospital for STEMI, likely due to atypical symptoms.^12^ It may be that this later presentation may have selected for a sicker patient at presentation, again making the interventions necessary prior to CCL transport more difficult. It may be that atypical symptoms (a lack of pain) gave the receiving emergency physician or CCL cardiologist pause, or that women presented with more STEMIs at the time of night during which the CCL team had to be called into the hospital. These findings point to needs for future study

## LIMITATIONS

We report results from a single, suburban, regional center with a high performance EMS agency transporting to a single receiving hospital, which requires consideration before any generalization of these results. The EMS agency has a long history of utilization and reliable interpretation of prehospital 12-lead EKGs. The combination of these factors made this process change more easily accepted at our institution than it may be in institutions where this historical working relationship is not in place.

During the data collection process, the specific emergency physician receiving the alert from either method was not recorded. Theoretically, if a specific physician or group of physicians decided to withhold or delay activation based on the method of notification this could induce bias and exaggerate the difference measured between the two methods. However, in the area of the ED that received the STEMI notification, there are anywhere from 5–7 different physicians working simultaneously and receiving the reports based on staffing needs. These physicians change every eight hours and are part of a group consisting of approximately 100 different physicians, which should provide a nice randomization.

It is also possible that a bias in patient randomization occurred as the paramedic’s decision to use the dispatch-aided method or the standard communication method was not a controlled decision. It is theoretically possible that slower paramedics consistently chose to use the standard communication method, making the standard communication rate seem falsely high. We did not collect paramedic identification data in this study limiting our ability to report on this possible bias.

False activation of the CCL remains a concern for any system employing paramedic activation of the CCL. While this study did not evaluate false activation, it is theoretically possible that removing the physician’s ability to discuss the case with the paramedic over the radio may increase the overall false activation rate. Anecdotal reports do not indicate this happened at our institution, but concurrent research is ongoing.

An additional limitation exists with respect to the methods of data abstraction. We did not perform interrater reliability testing as only one unblinded chart abstractor was used in addition to the blinded research nursing staff, and there was no oversight of this data abstractor.

## CONCLUSION

Early notification of STEMI by a 9-1-1 dispatcher-aided method achieves earlier CCL activation compared to notification by standard, direct communication from paramedic to physician in a hospital system that already uses EMS-directed CCL activation. This practice significantly decreased DTB by an average of 11.5 minutes per patient (95% CI [6.06,16.94]) and allowed a significantly higher percentage of patients (92% vs. 64%) to meet the DTB≤60 min metric. The intervention adds no cost or new technology, requires little training and requires only minimal changes to paramedic work processes.

We believe systems looking for additional ways to shorten DTB should consider this process.

## Figures and Tables

**Table 1:** Comparison of patient demographics by Notification Method, dispatcher-aided (new) vs. medic to physician (standard method), for ST-elevation myocardial infarction alerts.

Variable	New method	Standard method	p-value*
Age			
Mean (standard error)	61.0 (1.7)	64.4 (1.7)	0.16
Minimum (maximum)	30 (94)	34 (90)	
Sex (n%)			
Male	41 (64.1)	48 (75.0)	0.18
Female	23 (35.9)	16 (25.0)	
Race (n%)			
Asian	0 (0.0)	1 (1.6)	0.79
Black	5 (7.8)	3 (4.7)	
Hispanic	1 (1.6)	2 (3.1)	
Indian	-	1 (1.6)	
Unknown	1 (1.6)	-	
White	57 (89.1)	57 (89.1)	
Patients with coronary artery disease (n%)	16 (25.0)	16 (25.0)	1.0
Patients with congestive heart failure (n%)	1 (1.6)	4 (6.3)	0.36
Patients with atrial fibrillation (n%)	3 (4.7)	5 (7.8)	0.72
Patients with prior myocardial infarction (n%)	6 (9.4)	12 (18.8)	0.13
Patients with hypertension (n%)	37 (57.8)	38 (59.4)	0.86
Patients with diabetes (n%)	11 (17.2)	10 (15.6)	0.81
Patients with hyperlipidemia (n%)	25 (39.1)	31 (48.4)	0.28
Patients with prior stents (n%)	11 (17.2)	19 (29.7)	0.10
Prior catheter patients without stents (n%)	4 (6.3)	4 (6.3)	1.0

* P-values are calculated with a pooled t-test, chi-squared test or Fisher exact test when appropriate.

New method=medic-to-dispatch, standard method=medic-to-physician.
